# “I called people to carry me to the latrine”: Podoconiosis patients’ access to water, sanitation, and hygiene in Butaro, Rwanda

**DOI:** 10.1371/journal.pntd.0014158

**Published:** 2026-04-30

**Authors:** Nina Sandra Natasha Ngowi, Pacifique Ufitinema, Maria Albin Qambayot, Janna M. Schurer

**Affiliations:** 1 Center for One Health, University of Global Health Equity, Butaro, Rwanda; 2 Department of Infectious Disease and Global Health, Cummings School of Veterinary Medicine at Tufts University, North Grafton, Massachusetts, United States of America; University of Glasgow School of Life Sciences, UNITED KINGDOM OF GREAT BRITAIN AND NORTHERN IRELAND

## Abstract

Podoconiosis is a preventable Neglected Tropical Disease (NTD) that results in significant lower limb swelling and chronic disability. Its management can be done at home by regularly washing feet with clean water and soap and using protective footwear. This study aimed to explore podoconiosis patients’ experiences with access to water, sanitation, and hygiene (WASH) and to identify barriers and facilitators of home-based management (HBM). In this qualitative phenomenological study, participants were recruited following a screening conducted in Butaro Sector (Rwanda) to identify individuals with lower limb lymphedema. Randomly selected individuals underwent a clinical assessment, and only those confirmed to have podoconiosis were invited for an interview. In-depth interviews, supplemented by observation and photographs, were conducted. Transcripts were inductively coded using Dedoose (version 9.0.80) and analyzed through thematic analysis. Overall, 26 interviews were conducted across all five cells of Butaro Sector. Poverty and physical discomfort were major barriers in accessing WASH and HBM essentials, with many relying on family or neighbors for support. Shame, laughter, and discrimination from community members further hindered access to WASH. However, some participants found a financial solution in selling animal and household waste. Moreover, participants demonstrated little or no knowledge about podoconiosis which influenced their treatment choices. Many attributed the disease to witchcraft, blood infection, or God’s will. Common care choices included traditional healers, self-care, or health centers. These findings demonstrate the need to integrate community education into podoconiosis management programs to address misconceptions and stigma while promoting income-generating activities to ensure consistent access to HBM essentials.

## Author summary

In this study, we explored barriers and facilitators for home-based management (HBM) of podoconiosis, a Neglected Tropical Disease (NTD) caused by prolonged barefoot contact with irritant volcanic soils. Podoconiosis, characterized by lower limb swelling, pain, and chronic disability, disproportionately affects subsistence farmers. Additionally, stigma and widespread misconceptions about its causes and treatment hinder proper care. However, effective HBM that includes daily foot hygiene and protective footwear can alleviate symptoms and prevent disease progression. Using a qualitative phenomenological approach, we conducted in-depth interviews with 26 clinically diagnosed podoconiosis patients in Butaro, Rwanda. This was complemented by household observations and photography. Poverty, limited knowledge, and community stigma were major barriers to HBM. Social support facilitated access to WASH. These findings highlight the importance of integrating community education, stigma reduction, and economic empowerment into podoconiosis management, ensuring a comprehensive approach that addresses both patient needs and the broader challenges of disease prevention and care.

## Introduction

Podoconiosis is a chronic, non-infectious Neglected Tropical Disease (NTD) caused by prolonged skin exposure to irritant volcanic soils [1,2]. Early symptoms include burning sensations, itching, or discomfort of the foot, and mild toe and foot swelling [3,4]. As the condition progresses, symptoms can include severe leg swelling, thickening of the skin, formation of fibrotic nodules, and joint rigidity [5,6]. Podoconiosis patients may also suffer from acute dermatolymphangioadenitis attacks (ADLA), characterized by severe inflammation, intense fever, and a burning sensation in the lower limbs, that often leave individuals immobile and bedridden [5,6]. Podoconiosis predominantly affects subsistence farmers with limited access to water, sanitation, and hygiene (WASH) and those who are genetically predisposed [7–9]. While both men and women are affected, women in some contexts are more vulnerable due to social factors such as reduced access to footwear [10].

In Rwanda, podoconiosis is endemic in all districts, with a national prevalence of 68.5 cases per 100,000 people [11]. In 2017, an estimated 6,429 individuals were affected, primarily in highland districts such as Nyamasheke, Musanze, and Burera [11]. Podoconiosis imposes severe physical, psychological, and financial burdens on affected individuals, mainly due to chronic physical disability [12]. The increased size and weight of the legs make it challenging to perform household tasks or to work, reducing income on average by 37.3% [13]. The visible disfigurement caused by podoconiosis contributes to high levels of depression and social exclusion that can lead to missed opportunities for marriage, healthcare, education, and employment exclusion [6,14]. Podoconiosis patients from Musanze and Burera districts reported 19.8 times higher odds of depressive symptoms, and 10.7 times higher odds of anxiety compared to their healthy neighbors, mainly due to stigma and low self-esteem [15]. Family members of podoconiosis patients reported 1.5 times higher odds of experiencing severe anxiety symptoms than their healthy neighbors [15].

Podoconiosis progression can be prevented and partially managed through a simple and cost-effective approach of home-based management (HBM), which includes the following steps: (1) soaking feet daily in antiseptic water, (2) scrubbing between skin folds and toes; (3) applying emollients to prevent skin cracking; (4) elevating legs; and (5) performing exercises to improve circulation, decrease lower limb swelling, and lower the risk of ADLA attacks [6,14,16–19]. Such steps can be supplemented by bandaging limbs to reduce swelling, wearing closed-toed shoes, shifting to low-risk occupations, and replacing dirt floors to reduce soil exposure. Despite its simplicity, HBM can be challenging in contexts such as Ethiopia and Rwanda where access to clean water, healthcare support, essential medical supplies, and patient knowledge can be limited [20,21]. Moreover, low inclusion in medical and nursing curricula has meant that patients are often late to be diagnosed or to be informed about HBM [22].

The Government of Rwanda initially aimed to eliminate podoconiosis by 2024, targeting a prevalence of less than 1% through prevention, awareness, and self-management [23]. This target was not achieved. As of 2025, the country has extended its timeline aiming for elimination by 2030, reflecting the ambition of the effort but also the persistent barriers to delivering preventive and management measures equitably [24]. Improved WASH access is a key component of this plan, as skin and wounds should be cleansed with clean water to minimize progression and prevent the risk of secondary infections [25,26]. However, rural Rwanda remains underserved [27]. According to the National Institute of Rwanda (NISR) in rural Rwanda in 2022, 33% lacked access to safe water, and 21% of the population had no improved basic sanitation [28]. These figures highlight existing barriers faced by communities in these rural areas, including podoconiosis patients, for whom consistent foot hygiene and wound care are essential [29]. Moreover, little is known about how podoconiosis patients navigate these WASH challenges or the reasons for HBM non-compliance [30]. Addressing this gap requires methods that capture the lived experiences of podoconiosis patients. A qualitative approach is therefore well-suited to explore podoconiosis patients’ experiences in managing podoconiosis in their daily lives. Placing patients’ perspectives at the center of program design, implementation, and policy development aligns with the global NTD framework, which emphasizes community-driven strategies for sustainable elimination.

Podoconiosis is a One Health challenge as it lies at the intersection of human, animal, and environmental health. Individuals tending animals barefoot face elevated risk due to prolonged exposure to volcanic soil during grazing [31–33]. Animal activity near water sources can also cause environmental contamination, reducing water safety for human use, and undermining hygiene practices, which in turn exacerbates podoconiosis and other skin conditions [33–36]. These interconnections illustrate the need for integrated One Health strategies that simultaneously address human exposure, environmental conditions, and animal health [30,35].

Therefore, this qualitative study aimed to characterize the One Health barriers and facilitators that rural podoconiosis patients experience in practicing HBM and understand their access to essential supplies. Altogether, these results provide a basis for policymakers and healthcare providers to improve community-based services for rural podoconiosis patients.

## Methods

### Ethics statement

This research was approved by the Institutional Review Board at the University of Global Health Equity (UGHE-IRB 2024/316). The research team also sought and obtained approval from the Butaro Sector executive office (Ref13.4307.04.04.02). Before data collection, all participants provided written informed consent to be interviewed and photographed. No identifiable photographs were taken. To ensure confidentiality, each participant was assigned a unique identification number, and all photographs and transcripts were de-identified. All data were deleted from data collection devices immediately after transfer and securely stored in a restricted-access Google Drive folder accessible only to the research team.

### Study setting

This study was conducted in the Butaro Sector, Burera District, Northern Rwanda, which ranks fourth in Rwanda for podoconiosis cases, following Nyamasheke, Rusizi, and Musanze districts [11]. Butaro Sector is characterized by its hilly terrain and rich volcanic soils, which make it favorable for podoconiosis [11]. Agriculture is the primary economic activity, with 78.1% of households involved in farming and 56.7% of households involved in livestock [37]. Half the Butaro population lives below the national poverty line, which is defined as an income insufficient to meet basic needs, including food, healthcare, education, and shelter [38]. Access to WASH services is limited, with 37% of the population spending over 30 minutes to obtain water for humans and animals [27,29]. Additionally, 43.2% of households in Butaro use unimproved water sources, and 8.2% of households have access to unimproved sanitation facilities [29].

### Study design and data collection tools

This qualitative study design used a phenomenological approach to understand the lived experiences of podoconiosis patients regarding their access to WASH and HBM essentials. Study tools included a semi-structured in-depth interview guide and observation/ photography checklists of the WASH and HBM essential items used within their homes. The interview guide was adapted from previous studies on lived experiences and modified for the Rwanda context [39,40]. It contained ten questions, each supplemented with additional probes designed to gather in-depth information aligned with One Health barriers and facilitators to HBM and WASH access. It was then revised after consultation with two independent researchers with extensive expertise in qualitative health research and podoconiosis-related studies. Their input focused on ensuring that the guide was clear, culturally appropriate and comprehensive. Based on their feedback, we revised wording for simplicity, reorganized question flow, and added probes to encourage more in-depth responses. We then translated it from English into Kinyarwanda. The observation and photography checklists focused on key WASH indicators such as household water sources, sanitation facilities, floor types, and hygiene essentials such as soap, basins, or handwashing stations at participants’ homes. The three tools were pretested on five podoconiosis patients outside the study area. Feedback from pretesting helped in creating a more comprehensive and contextually relevant guide and checklists.

### Participant recruitment and sampling

To recruit participants, Community Health Workers (CHWs) from all 68 villages of Butaro Sector first visited all households to identify individuals with lower limb swelling. To characterize the swelling and its relevant history for each identified individual, CHWs completed podoconiosis checklists with the following five criteria: (1) walks or works barefoot in soil daily; (2) swelling of both legs below the knees; (3) family history of bilateral leg swelling; (4) swelling present for more than one year; and (5) swelling that does not resolve overnight (S1 Checklist). Next, we categorized possible cases into three groups: “highly probable” (met all five criteria, “probable” (met four), and “not a case” (met three or fewer). The highly probable and probable cases were compiled into a final list, disaggregated by gender, and assigned random numbers using a computer-generated sequence in Excel. This random list formed the basis for participant selection. Selected individuals were visited at home by two research team members, including a medical doctor (P.U.) who clinically examined them to confirm podoconiosis before inclusion. Those identified as podoconiosis cases, at least 15 years of age, and with a minimum 1-year residence in Butaro were invited for an interview. Those suffering from any mental health disorder that could hinder them from participating in the study were excluded.

Sampling was guided by the principle of data saturation, with an initial target of 26 interviews, consistent with other studies that explored the same phenomenon [41]. Interviews were analyzed concurrently, and data collection continued until no new codes emerged in three consecutive interviews. For instance, if a visited individual was not a podoconiosis case, the next person on the randomized list was approached until data saturation was achieved. Once no new codes emerged, the research team conducted four additional interviews to confirm meeting saturation. This approach is consistent with established methodological guidance showing that qualitative studies often reach saturation within 12–24 interviews, with further interviews strengthening conceptual richness [42,43].

### Data collection

In-depth interviews were conducted in Kinyarwanda, at participants’ homes, and audio recorded. Podoconiosis severity was evaluated using the Tekola clinical staging system [42]. And participants’ homes were photographed (including sanitation facilities, water sources, flooring, and hygiene items). At the end of each interview, participants received information about podoconiosis as well as basic hygiene supplies for HBM.

### Data analysis

Audio recordings were transcribed verbatim and translated into English by five native language speakers. Analysis followed an inductive approach informed by phenomenological principles [44]. To enhance reflexivity, the research team discussed their own assumptions prior to coding to minimize the influence of preconceptions on interpretation. Transcripts were read openly to allow immersion in participants’ narratives, and an initial codebook was developed after reviewing the first ten transcripts. Two researchers (P.U. and N.N.) coded all transcripts independently, then met to compare codes and resolve any discrepancies. Coding was performed inductively in Dedoose software (Version 9.0.86) which facilitated organization of codes and supported reduction by clustering descriptive codes into broader categories that captured the essence of participants’ lived experiences. Data visualization tools (e.g., code co-occurrence and frequency displays) were also used to observe connections and patterns among the codes and the developing themes, but interpretation remained grounded in participants’ own words and meanings.

## Results

### Participants’ screening results

**Fig 1:**
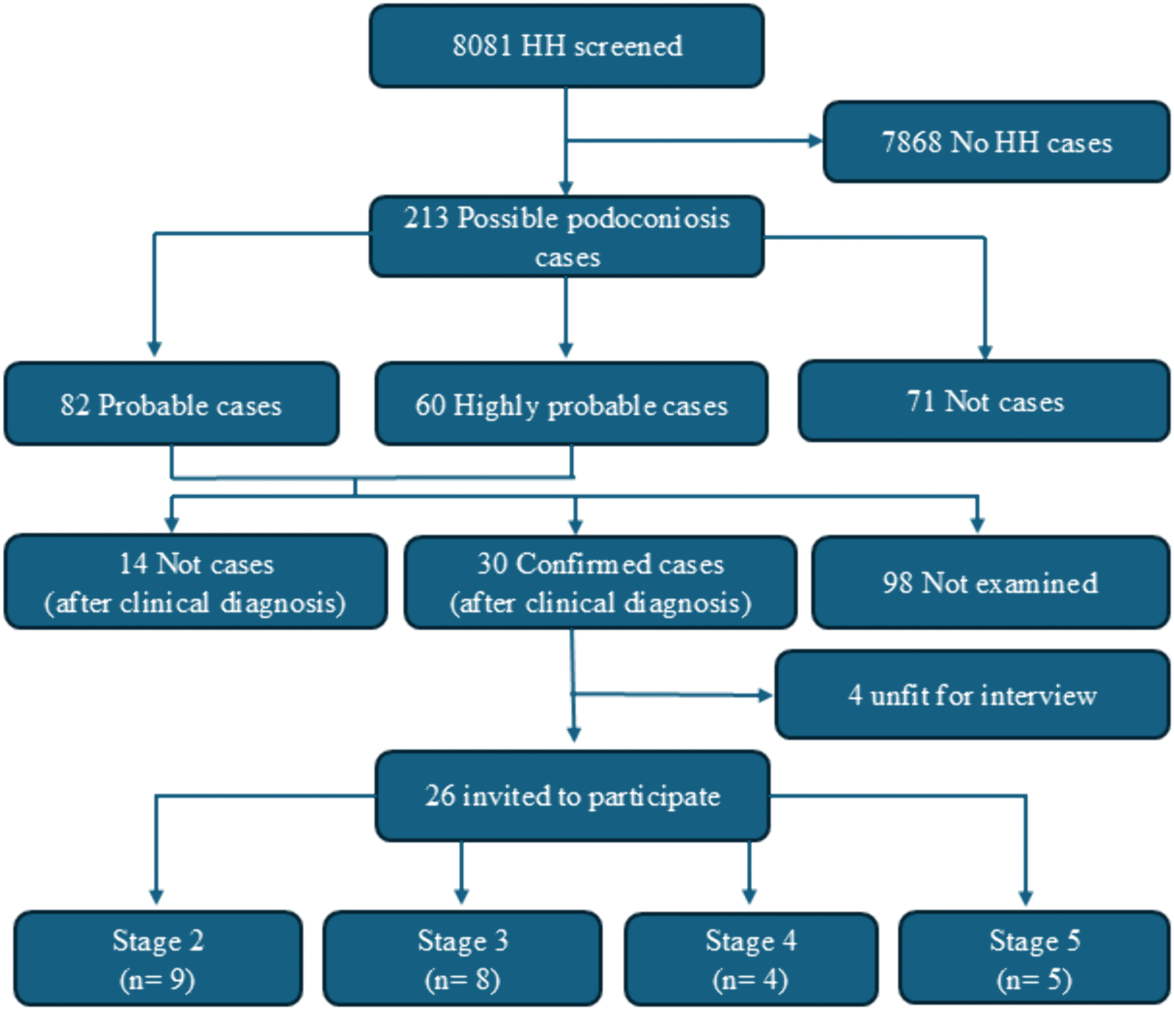
Podoconiosis screening, diagnosis, and staging in Butaro Sector, Rwanda.

**Fig 2:**
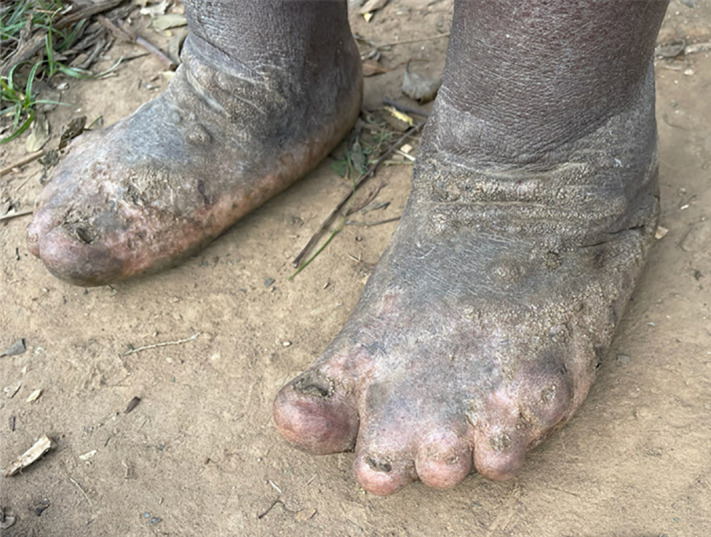
Podoconiosis patient with Stage 3 in Butaro Sector, Rwanda.

CHWs screened 8081 households in the Butaro Sector and identified 213 possible podoconiosis patients (Fig 1). Clinical examination and staging of the first 44 cases confirmed podoconiosis in 30 (Fig 2). Of these, 26 individuals were invited to participate in in-depth interviews; the remaining four were deemed mentally unable to partake.

### Participant demographics

Of the 26 participants, 16 were female (61.5%; Table 1) and two-thirds fell within the 50–99 year age range. The mean participant age was 55.3 years, with a standard deviation (SD) of 19.1 years. Farming was the main occupation for participants (53.8%). Those with animals (57.7%) owned cattle, sheep, pigs, chickens, goats, and rabbits.

**Table 1:** Demographics and Tekola stages for podoconiosis patients in the Butaro Sector.

Variable	Count (n)	Percentage (%)
Sex		
Male	10	38.5
Female	16	61.5
Age group (years)		
15-49	9	34.6
50-99	17	65.4
Households with animals		
Yes	15	57.7
No	11	42.3
Tekola Stages		
Stage 1	0	0
Stage 2	9	34.6
Stage 3	8	30.8
Stage 4	4	15.4
Stage 5	5	19.2

### WASH observation results

**Fig 3:**
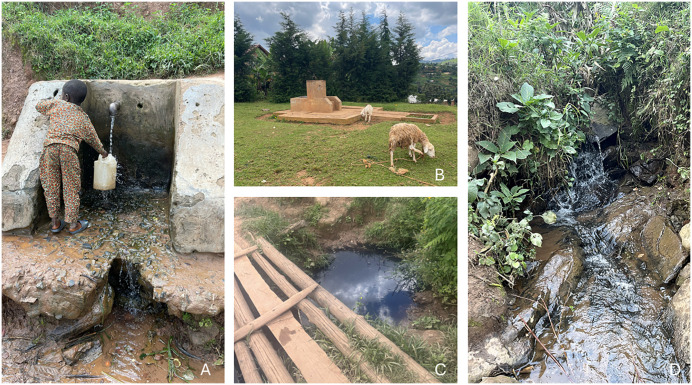
Types of water sources used by podoconiosis patients in Butaro Sector, Rwanda. **A**: Tube well, 40 minutes’ walk from the patient’s home. **B**: Tap water, 5 minutes from the patient’s home, but operates 1x/week. **C**: Pond water, less than 5 minutes from the patient’s home. **D**: Spring water, 20 minutes away from the patient’s home.

**Fig 4:**
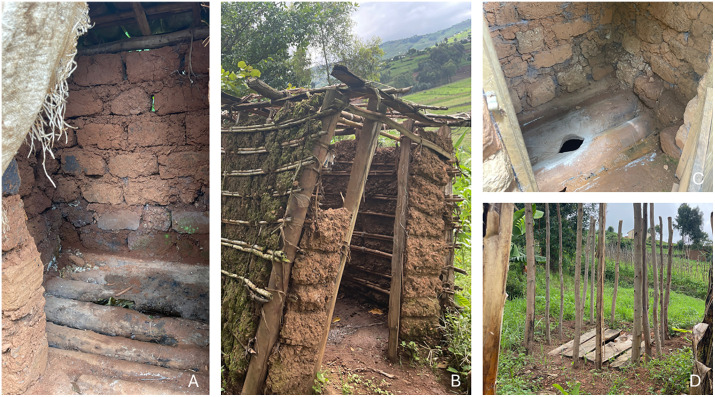
Latrines used by podoconiosis patients in Butaro Sector. **A**: One of the poorly maintained latrines. **B**: An almost falling toilet. **C**: The cleanest pit latrine observed. Participants used ash (white powder) for cleaning and reducing smell. **D**: Unfinished but used latrine.

**Fig 5:**
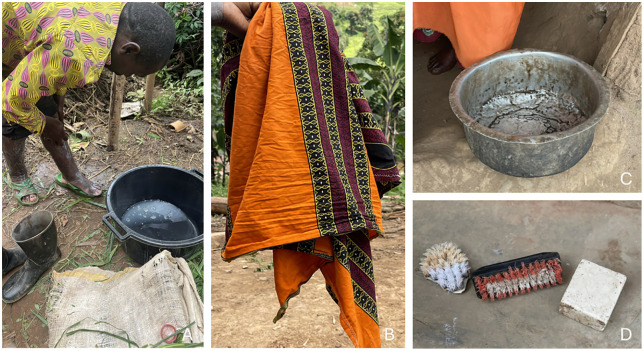
Items used by participants in podoconiosis HBM. **A:** One of the cleanest basins used by the participants. **B:** “Kitenge” used in place of towel. **C**: Saucepan used to wash feet. **D**: Various HBM items (soap and brushes) used by participants.

**Fig 6:**
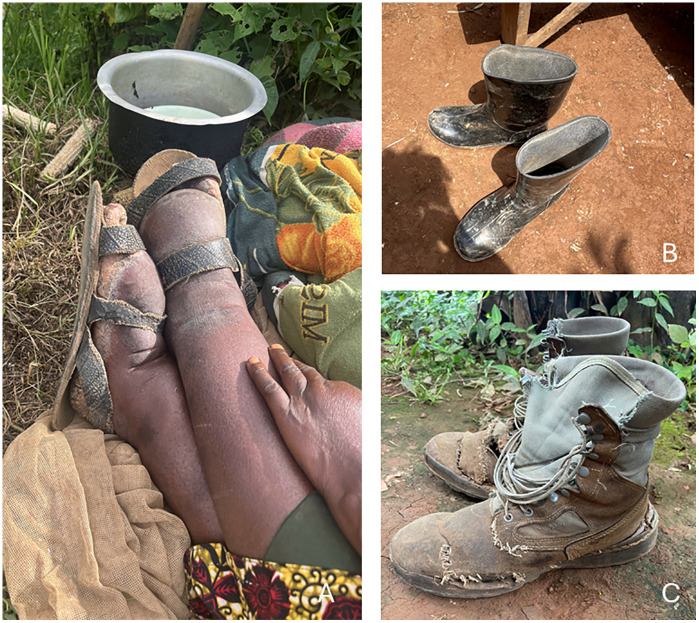
Shoes worn by podoconiosis patients in Butaro Sector, Rwanda. **A**: Rugabire shoes. **B**: Plastic boots. **C**: Work boots.

No participants had water sources at home. Public sources, including tube wells, tap water, spring water, and ponds, were reached within 40 minutes of walking (Fig 3). Almost all households had uncovered pit latrines, with some being clean and well-maintained while others were dirty, lacking doors, or falling apart (Fig 4). Most participants had essential items for HBM, such as cut jerrycans, saucepans, and basins to wash their feet, but used kitenge (African wrap cloth) rather than a towel to dry their feet after washing (Fig 5). Most households had uncovered mud floors; the rest had concrete. At the time of the visit, most participants were wearing shoes (Fig 6).

### Themes

Participants’ narratives revealed how living with podoconiosis shaped everyday experiences, illness interpretations, hygiene practices, and social relationships. The findings reflect how participants made sense of their condition, navigated WASH practices within physical, economic, and social constraints, and adopted adaptive strategies to sustain their daily life. These four interrelated themes emerged from the analysis:

1. Podoconiosis within socio-cultural and policy contexts.
2. Physical abilities, environmental conditions and WASH.
3. Economic barriers and stigma.
4. Facilitators of access to WASH and HBM essentials.

#### Theme 1: Podoconiosis within sociocultural and policy contexts.

**Subtheme 1: Beliefs and illness interpretations:** Beliefs and illness interpretations shaped podoconiosis patients’ self-care practices and health care seeking. Living with podoconiosis was experienced as a journey of making sense of unfamiliar body changes, where participants relied on their beliefs or cultural interpretations to understand their illness. Most of them had little knowledge about podoconiosis causes, at-risk populations, or treatments. They attributed podoconiosis to witchcraft, environmental substances like crane droppings, a local urinary tract infection known as “Ifumbi,” or God’s will. Care-seeking pathways reflected these interpretations. Some participants sought help from traditional healers or pursued hospital treatments, which included injections, ointments, and pills, providing minimal pain relief without reducing swelling, while others opted for self-care, such as soaking their feet in hot water. For them, these beliefs were not mere abstract ideas, but real explanations that shaped how they understood their illness, assessed the treatment effectiveness, and the decision on the choice of care and relief.

*“I think podoconiosis is a disease of witchcraft. When I started getting sick, a friend of mine recommended that I wrap the tail of the polecat on my legs, which I did for one year. I stopped it when I lost my tail. It helped me with the pain for a while, but the swelling did not go away*.” ***(Female, 44 years old)***

*“When I started experiencing leg swelling, I went to a woman who treats such kind of diseases in our village, and she gave me herbs to wrap around my legs. I used them for a year then stopped because they were not helping me. My neighbor told me to soak my feet in hot water before going to sleep. I always do it, and I feel better when I wake up in the morning.” (****Female, 56 years old***)

*“After realizing that my legs were swollen, I went to [an International Health NGO]. They gave me 100 pills to put in one litre of hot water and recommended soaking my feet in that water. I did it, but it didn’t change anything. I also used some herbs, which also did not help me. Then I stopped everything and accepted my condition, trusting that only God would heal me.”* ***(Female, 62 years old)***

**Subtheme 2: Comfort, protection, cultural norms, and government policy:** Cultural norms, government policy, comfort and protection collectively encouraged shoe-wearing as both protective practice and a social obligation. Most participants began wearing shoes in public spaces when a government policy mandating shoe-wearing in Rwanda was introduced in 2010. They acknowledged shoes as protective against dust and stones, but also mentioned discomfort and worry about aging, loss, or wearing out, as finding new, fit-friendly shoes in larger markets was challenging. In this way shoe use emerged as an ongoing negotiation between body comfort, health protection, and social acceptability.

*“Most of my life, I did not wear shoes. I started wearing shoes because we were recommended to do so. It was Kagame* [President of the Republic of Rwanda] *who said that no Rwandan should walk barefoot. However, my legs continued to increase in size, and I was not able to get shoes. One day, I saw people making sandal shoes called Rugabire* [shoes made from old car tires]*. They took measures of my feet, and they made sandals for me. From that time, I never removed them.”* ***(Male, 58 years old)***

*“I mainly worked barefoot for a long time because working in that mud, mixing it, and making bricks is not easy with shoes, but with this disease, I cannot go anywhere without shoes because there are stones that disturb me.”* ***(Female, 44 years old)***

*“During farming, I used to remove my boots and wear them again after fieldwork because it was uncomfortable. The soil gets stuck on them, making it hard to move my legs. However, I am no longer able to do farming. I stay here. That’s why I am wearing shoes.”* ***(Male, 95 years old)***

#### Theme 2: Physical abilities, environmental conditions and WASH.

**Subtheme 1: Physical strength and functional capacity:** Participants’ physical strength affected their ability to perform daily WASH-related activities. As symptoms progressed, normal movements became physically demanding, turning routine tasks into sources of pain. Participants with severe symptoms, such as increased pain, leg swelling, and weakness, struggled to complete even simple tasks such as walking to the water source. This sometimes left them without water for an entire day or caused them to avoid work and limit their use of latrines. Those with fewer symptoms handled daily activities such as farming, tending to animals, and doing other household chores such as cooking or waste management. Loss of strength was often experienced as a loss of independence, with simple acts such as walking to a latrine becoming a daily reminder of illness and dependency

“There was a day when my symptoms got worse, and my legs were huge. For me to move anywhere I needed people to carry me. So, what I did was stop eating so that I didn’t need to go to the latrine. It was embarrassing to call people to carry me to the latrine every time I needed to go there.” **(Female, 26 years old)**

“I am no longer able to walk to the water source. I am not even able to cultivate on my farm here. How can I do it with these legs? As you can see, I am worthless. My grandchildren fetch for me. If they refuse, I can even spend a day without water.” **(Male, 62 years old)**

**Subtheme 2: WASH infrastructures:** Participants’ water sources fell into 2 categories: (1) fee-for-service water sources (nearby, clean, sometimes operational), and (2) free water sources (1–2 hours walk, unclean, always available). Most had latrines near their homes, but some lacked functional ones, forcing reliance on neighbors’ latrines or open defecation. Small hygiene essentials, such as soaps and emollients, were readily available, while larger items such as shoes, basins, and towels were only found in distant markets. Accessing water, obtaining hygiene essentials and using latrines required long journeys that were described as not only exhausting but also isolating, as mobility challenges turned routine tasks into daily struggles.

*“In this region, we have two water sources. There’s a tap nearby, but it requires payment, so I rarely go there. For us who don’t always have money, there is a surface water source that is one hour from here, and that is where we go. It is hard for me to always go there because whenever I go there, my legs worsen.”* ***(Male, 41 years old)***

*“My latrine collapsed a few weeks ago... During the day I use my neighbor’s toilet, while at night I use a small pot or go to my crop fields. I can’t lie to you.”* ***(Female, 75 years old)***

“When we need to buy a new basin or shoes, we go to Nyamicucu market. We don’t have a market here. I think it would be easier for us if we had a market around here. Nyamicucu is so far.” **(Male, 16 years old)**

**Subtheme 3: Seasonal variations:** Seasonal variations shaped how participants felt in their bodies and when they were able to carry out daily activities. Some participants experienced symptom relief during hot or dry seasons, with softer, less painful legs and reduced swelling. Other participants reported improved symptoms in the rainy season or cold weather, preferring to complete activities in the morning when they felt better. For some, rainwater facilitated water access, reducing the need for long walks to distant water sources. Others found muddy roads difficult to navigate. Seasons were therefore experienced not only as shifts in weather but as changes in the body; where sometimes participants felt more capable and freer, or more constrained and vulnerable.

*“Here we live, we usually find it easy to get water, especially during the dry season. Even if water reduces during this season, it is always available.”* ***(Female, 44 years old)***

*“When it rains, my legs are soft, they don’t hurt me as much as they do when it’s sunny. And I can see that the swelling has decreased a bit. I like it when it’s cold because I am very active and can cultivate easily.”* ***(Male, 58 years old)***

#### Theme 3: Economic barriers and stigma.

**Subtheme 1: Poverty:** Poverty was a major barrier to accessing essentials for HBM across all participants. It extended beyond material deprivation and shaped daily decision-making. Participants described having to prioritize basic survival needs over hygiene and self-care, often making difficult trade-offs between purchasing soap, food or paying school fees. Basins, towels, and emollients were costly, and some water sources required payment, which they often could not afford. In addition, participants’ physical conditions made it difficult to earn income for basic needs such as food or hygiene items. To adapt, soap or shoes were used occasionally, feet were dried by air rather than by towel, and distant water sources were used. Of the HBM essentials, emollients and towels were the least purchased.

*“Due to poverty, sometimes we wash our bodies without soap. Some other days we have soap but no lotion, so we use the soap to soften our skin. As for the towels, we are used to air drying, making do with what we have.”* ***(Female, 44 years old)***

*“Due to my disease, I have not worked well recently on my small land. Most of the time, I go to sleep with an empty stomach. Soap is the last thing I can think of buying.”* ***(Male, 44 years old)***

**Subtheme 2: Stigma and discrimination:** Participants experienced exclusion and negative attitudes from their neighbors and family, which reduced accessibility to WASH and HBM essentials.

Women, in particular, faced mistreatment from their spouses, who denied them access to latrines and hygiene items, turned neighbors against them, and forced them to sleep outside. These experiences led to feelings of shame, low self-esteem, directly limiting participants’ ability to carry out daily self-care.

*“One day I went to fetch water, and I met there some ladies of my age. They started laughing at me, saying, ‘Look at that one with fat legs.’ I was hurt and felt ashamed. Sometimes I hesitate to go to fetch water again, but because I need water, I don’t have a choice. From that day, I go to fetch water early in the morning, like at 4 or 5 a.m., to be the first there, so that I don’t meet them again.”* ***(Female, 26 years old)***

*“I told you that getting soap is a struggle because my ex-husband went to the market and gave everybody who sells soap money, telling them to never give me soap, even if I paid them. He also refused to let me dig a latrine on his land. He said I don’t deserve a latrine. I am just surviving by the grace of the Lord.”* ***(Female, 50 years old)***

*“One day, I went to fetch water, but when I came back, I found that my ex-husband had taken everything from me. There is a basin that I recently bought; he also took that. I am currently using a saucepan to shower and wash my dishes.”* ***(Female, 44 years old)***

#### Theme 4: Facilitators of access to WASH and HBM essentials.

**Subtheme 1: Animal and household wastes:** Livestock manure, combined with other household waste, was sold to generate income, enabling participants to regain control and resilience in the face of poverty. Some had entered into agreements with neighbours to rear animals without owning livestock. This practice enhanced agricultural productivity and alleviated financial burdens by increasing their income from crop yields or manure sales. For many, they were more than financial strategies. There were ways of regaining control and resilience in the face of poverty.

*“I collect all the waste from my cow and goats, from cooking and sweeping then I mix them in the area where my cow stays so they can decompose together. After a few days, they turn into manure, which I sell to local farmers. A package of this fertilizer sells for six thousand Rwandan francs (=4$).”* ***(Female, 42 years old***)

**Subtheme 2: Social support:** Support from family members, community, and healthcare professionals positively influenced access to WASH and HBM essentials. Most participants had lived with podoconiosis for over 10 years, describing their experiences as a blend of challenges, resilience, and support from family, neighbors, and healthcare providers. This support included fetching water for older patients, purchasing their hygiene essentials, helping with household chores, providing financial aid, and supporting them morally. Participants expressed that such support restored dignity and a sense of belonging and eased the loneliness of living with podoconiosis.

*“My daughter-in-law came here and saw my legs; she immediately bought me a brush to use while washing my feet. Since then, she has always been here to make sure that I wash my legs properly.”* ***(Female, 68 years old)***

*“As you can see, I live here alone. I don’t have children, and I never got married. Our community health worker is the one who takes care of me. She sometimes buys me food and soap.”* ***(Male, 41 years old)***

## Discussion

This study shows that living with podoconiosis was experienced by participants as a daily struggle, shaped by bodily pain, restricted mobility, social exclusion, and dependence on others for care. Participants described how swollen and painful legs altered everyday routines such as fetching water, using latrines, and maintaining hygiene, while social responses ranging from ridicule to support profoundly influenced their ability to practice HBM. These lived experiences provide essential context for our understanding of gaps in access to WASH and HBM essentials. To our knowledge, this is the first study in Rwanda to contextualize the experiences of podoconiosis patients on access to WASH and essentials for HBM. Global health frameworks, such as the WHO NTD Roadmap 2030, increasingly emphasize the integration of community-driven perspectives into strategies for eliminating NTDs [45]. In Rwanda, the government is committed to improving community awareness about NTDs, including podoconiosis, while advancing a One Health approach for their prevention, control, and elimination as highlighted in the 2019–2024 national strategic plan for NTDs [23]. Additionally, Rwanda had pledged to achieve 100% access to WASH services by 2024 recognizing its key role in NTD elimination [46]. Despite this strong foundation, our study identified significant gaps between the policies’ intended outcomes and the reality at the community level. Study participants demonstrated limited knowledge about podoconiosis, community stigma, and inadequate access to WASH facilities. No dedicated programs exist to reduce disease risk, combat stigma, or improve patient outcomes. Altogether, this indicates an urgent need for equity-focused, multisectoral interventions to bridge the gap between national strategies and the realities faced by vulnerable communities. Our findings challenge the assumption that policy alone ensures progress, showing instead that awareness, access, and stigma remain major barriers undermining elimination goals [47].

Podoconiosis patients in this study demonstrated varied and often incorrect perceptions regarding disease etiology and treatments, attributing podoconiosis to witchcraft or harmful substances in the environment, such as poison or herbs. Similar incorrect beliefs, such as transmission occurring through contact with affected individuals or by touching the soil around cemetery graves, have been documented in Ethiopia, Cameroon, and Rwanda [45–47]. Moreover, these interpretations influenced how participants responded to symptoms and where they sought care, often prioritizing traditional remedies over formal medical care or not seek care at all. However, some participants also sought care from formal health providers, including non-governmental health organizations, but reported limited improvement in symptoms. Such experiences may contribute to frustration, reduced trust in formal medical care, and, eventually, reliance on traditional remedies [48]. In this study, some participants wrapped animal parts (such as tails) around their legs, while Ethiopian patients washed with holy water [49]. While education and training are emphasized, current campaigns may not be reaching those most affected [50]. These findings highlight the need for educational interventions targeting the community to bridge the knowledge gap, promote preventive practices, and encourage timely seeking behavior thereby improving the effectiveness of podoconiosis treatment. This would also involve training healthcare professionals to improve their knowledge about podoconiosis, as it is limited [21,50]. More locally tailored and culturally sensitive strategies are needed to ensure that prevention messages truly resonate with patients’ lived experiences [51].

Both externalized and internalized stigma significantly impacted participants’ access to WASH, manifesting through social rejection, discrimination, and isolation from their communities and families. Some participants were forbidden by their domestic partners to construct latrines, forcing them to use poorly maintained shared latrines, often barefooted, or resort to open defecation. Such practices expose vulnerable patients to co-infection with other NTDs, such as soil-transmitted helminths, and worsening patient outcomes [52]. Other participants were denied purchasing hygiene essentials from local shops or were ridiculed at water sources. To avoid this, they refrained from returning to those places, hid their legs with clothes, or chose to use less frequented roads. These experiences reshaped participants’ lives manifesting as feelings of humiliation and a diminished sense of belonging, which directly constrained WASH practices [53]. These behaviors were also observed in northwest Cameroon and southern Ethiopia, contributing to poor proper care and reduced opportunities for social and economic engagement [54–56]. Within the NTD framework, stigma reduction is increasingly recognized as a key factor of disease elimination [57]. Our findings support this by showing deeply stigma erodes belonging and limits patient’s ability to even practice basic hygiene [58]. Integrating stigma-reduction programs such as community dialogues and peer-led support groups into podoconiosis interventions is essential for improving social acceptance and advancing the effective management and prevention of podoconiosis [20,27,59]. For instance, in southern Ethiopia, community engagement initiatives have successfully reduced stigma and improved adherence to preventive measures such as wearing protective footwear and maintaining foot hygiene [20,27,59]. By contrast, the care of family, neighbors, and CHWs fetching water, providing items, restoring dignity brought relief and reassurance, enabling self-care despite bodily pain and spatial barriers [15,60]. Although podoconiosis patients in this study faced significant challenges, family members, neighbors, and CHWs facilitated access to WASH and provided support in daily tasks. This included fetching water, assisting with latrine use, caring for their animals, or offering hygiene supplies, food, and money. Most participants relied on this support, as they did not have a water source within their households and faced difficulties traveling long distances to fetch water. The inability to access water consistently hindered self-care practices, exacerbating their symptoms and increasing their risk of ADLA attacks. These findings align with studies in Ethiopia and Haiti, where the support of family and community networks played a role in overcoming barriers to WASH and reducing the burden of podoconiosis [19]. Moreover, interventions such as community-based treatment programs that improve access to water and essential foot care items have been shown to significantly reduce the frequency of ADLA attacks and improve podoconiosis patient outcomes [20,59,61]. In this way, our findings both support and extend NTD frameworks by showing how lived experiences of dependence and care are not just personal challenges but essential factors for tailoring interventions that work within communities [62]. The reliance on this support, particularly for individuals with limited physical mobility, highlights the necessity of strengthening community engagement and empowering support networks to play a more effective role in enhancing the overall quality of life for podoconiosis patients [60,59,63].

This study faced a few limitations such as recall and social desirability bias, but data validity and reliability were enhanced by using a triangulation approach, combining self-reported data with direct observations and assessments. For example, if a participant claimed to have a nearby water source, we visited the site to confirm its proximity and functionality. Similarly, participants were asked to show available hygiene items and sanitary facilities instead of relying solely on verbal information. This method ensured cross-checking of information, thereby enhancing credibility and trustworthiness [64]. Recall bias was a major challenge, as participants often struggled to accurately remember specific events or behaviors. To address this, we used probing questions to help participants reconstruct their experiences in greater detail. Social desirability bias was another limitation, where participants tailored their responses to align with perceived expectations, especially in the presence of family members. This was mitigated by creating a non-judgmental environment, ensuring confidentiality, and emphasizing that differing perspectives were welcome. Lastly, four individuals diagnosed with podoconiosis were excluded from this study due to congenital mental health disorders that prevented them from being able to provide informed written consent. While not formally investigated in the study, this observation suggests that podoconiosis is concentrated among individuals facing compounded disadvantages, including reduced capacity for regular foot care, social exclusion, and limited access to footwear and treatment. These factors may increase both the risk of disease and progression to more advanced stages and should be explored in future research. Despite these limitations, this study provides important insights into barriers to WASH and HBM essentials among people affected by podoconiosis in Rwanda, including poverty, stigma, distant water sources, and limited access to appropriate footwear. It also highlights social support from neighbors, CHWs, and family members as a key facilitator that enables access to WASH and supports self-care. These findings inform targeted, equity-oriented, community-driven interventions aligned with the NTD framework.

## Conclusions

This study highlighted how swollen, painful legs shaped daily decisions, how stigma undermined belonging, and how family or community support restored dignity and capacity. Altogether, this points to the utility of adopting a holistic approach that integrates 1) community education to dispel stigma and misconceptions; 2) economic empowerment initiatives to address poverty and ensure sustainable access to WASH; and 3) support networks to improve podoconiosis patients’ overall outcomes. Centering lived experience alongside structural interventions ensures strategies are not only effective but also meaningful to those most affected. Focusing on these community-centered solutions may help Rwanda make significant strides toward achieving its goal of eliminating podoconiosis. Moreover, the successful implementation of these interventions could serve as a model for other regions facing similar challenges, thereby contributing to the broader goals of reducing the burden of NTDs globally.

## Supporting information

S1 Checklist Podoconiosis screening checklist. (DOCX)
