# Supplementary material for: “I called people to carry me to the latrine”: Podoconiosis patients’ access to water, sanitation, and hygiene in Butaro, Rwanda
Source: PLoS Negl Trop Dis. 2026 Apr 30;20(4):e0014158. doi: 10.1371/journal.pntd.0014158 (PMC13132447; doi:10.1371/journal.pntd.0014158)
Supplement: S1 Checklist — (DOCX) [file pntd.0014158.s001.docx]

## **PODOCONIOSIS SCREENING CHECKLIST**

CHW name:

CHW contact (Tel):

Screening location: District:

Sector:

Cell:

Village:

NB: To fill this table use **“**✔**”** for a “Yes” and **“X”** for a “No” answer to the five listed criteria.

## For the possible/ probable case, will be left blank for the PIs to fill in.

Similarly, the box for “consent to be contacted” you will do the same. For the remaining boxes, fill them depending on the individuals’ answers.

Individuals with four to five criteria will be considered as possible/ probable cases of podoconiosis and will be the ones included in the study participants list.

**HH:** Represent the Household number


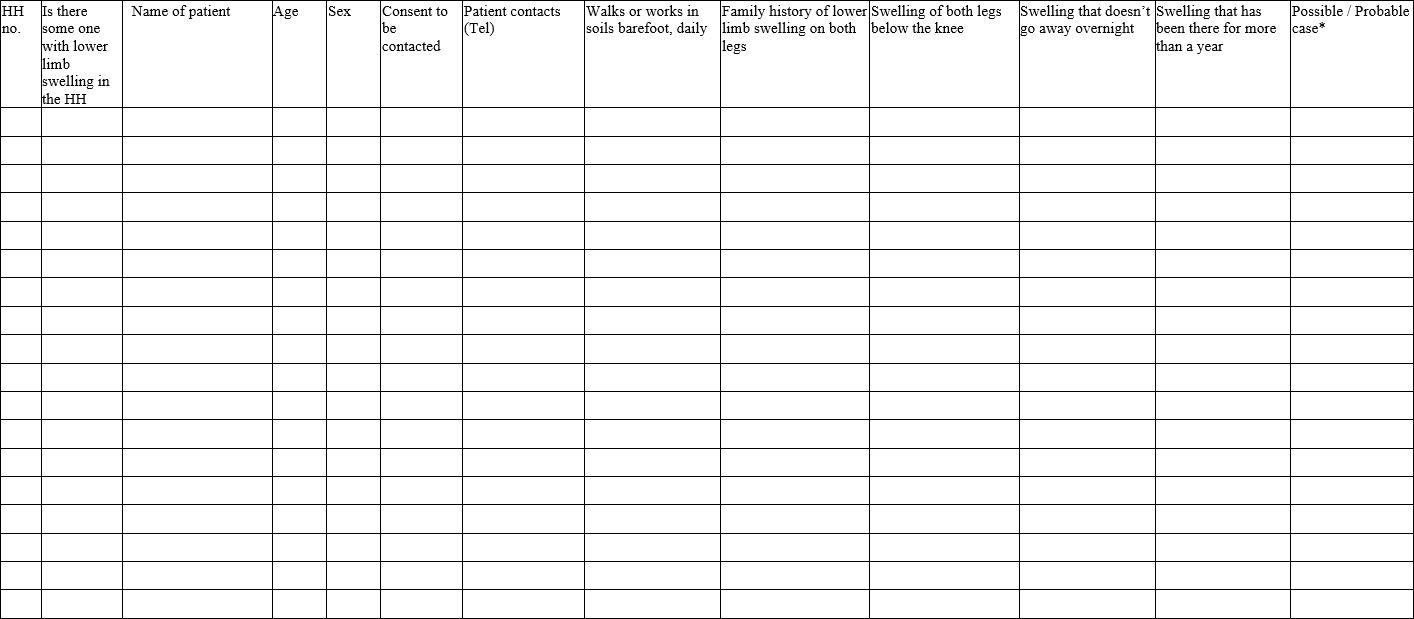


# **INTERPRETATION**

Based on the criteria outlined in this screening list, participants will be categorized as follows: Participants selected from the list will be confirmed as podoconiosis cases by {attending physician} prior to being invited to participate in this study. S/he will use the podoconiosis diagnostic protocol described below.

This protocol is based on a validated diagnostic tool extensively used for identifying podoconiosis cases through clinical evaluation. The diagnosis will be approached systematically, ensuring all criteria are evaluated comprehensively to avoid misdiagnosis.

Step 1: Initially a case assessment will be conducted by taking a detailed patient history followed by a physical examination of the legs.

Step 2: The assessment will be followed by diagnosis confirmation referring to the following criteria

## Major Criteria

- Presence of lower limb swelling below the knee.
- Prolonged barefoot exposure exceeding 10 years.
- Residency in a podoconiosis-endemic area coinciding with the onset of symptoms.
- The presence of ‘mossy foot’ characterized by a slipper-like pattern.

## Minor Criteria

- Family history of podoconiosis.
- Onset of symptoms within the first three decades of life.
- Experience of any burning sensations in the affected limbs.
- Presence of nodules.
- Bilateral foot involvement.
- Fusion of toes.

A case of podoconiosis will be confirmed in case a person has either one of the following combinations:

1. Three major criteria.
2. Two major criteria + two minor criteria.
3. One major criteria + five minor criteria.
